# PD-1/PD-L1 inhibitor treatment and its impact on clinical imaging in non-small cell lung cancer: a systematic review and meta-analysis of immune-related adverse events

**DOI:** 10.3389/fonc.2023.1191681

**Published:** 2023-09-29

**Authors:** Nader Mohammed, En-Hua Xiao, Shallal Mohsen, Zeng Xiong, RongRong Zhou

**Affiliations:** ^1^ Department of Radiology, The Second Xiangya Hospital of Central South University, Changsha, Hunan, China; ^2^ Diagnostic Radiology Department, Cairo University, Cairo, Egypt; ^3^ Xiangya Lung Cancer Center, Xiangya Hospital, Central South University, Changsha, Hunan, China; ^4^ Department of Radiology, Xiangya Hospital, Central South University, Changsha, Hunan, China; ^5^ Department of Oncology, Xiangya Hospital, Central South University, Changsha, Hunan, China; ^6^ National Clinical Research Center for Geriatric Disorders, Xiangya Hospital, Central South University, Changsha, Hunan, China

**Keywords:** imaging, lung cancer, PD1/PDL1inhibitor, adverse events, non-small cell

## Abstract

**Background:**

In the contemporary era of cancer treatment, lung cancer (LC) holds the unenviable position of being the primary contributor to cancer-induced mortality worldwide. Although immunotherapy has expanded the therapeutic landscape for metastatic non-small cell lung cancer (NSCLC), the advent of immune checkpoint inhibitors has been accompanied by a concomitant increase in immune-related adverse events (irAEs). Timely detection of irAEs is pivotal for efficacious management and enhanced patient outcomes. Diagnostic imaging, encompassing x-ray and CT scans, can facilitate the identification and supervision of irAEs, thereby ensuring the prompt recognition of associated patterns and alterations for expeditious treatment.

**Methods:**

The present inquiry undertook a systematic exploration of multiple databases, incorporating a diverse array of studies such as randomized controlled trials and observational analyses. Patient demographics, imaging outcomes, and risk of bias were extracted from the data. Meta-analysis was executed utilizing R Statistical Software, with the results of the risk of bias assessment summarized accordingly.

**Findings:**

The analysis unveiled a higher prevalence of irAEs in patients receiving first-line treatment for NSCLC compared to those receiving subsequent treatments, with a statistically significant distinction observed for both high- and low-grade irAEs (p < 0.001). Pneumonitis, thyroiditis, and colitis emerged as the most frequently reported irAEs, whereas hepatitis and pancolitis were less commonly documented. This investigation signifies a crucial advancement in elucidating the function of imaging in the treatment of NSCLC with PD-1/PD-L1 inhibitors and emphasizes the imperative for ongoing research in this domain.

## 1 Introduction

Lung cancer is the main cause of cancer-related death globally, and novel therapeutic approaches are required to enhance patient outcomes. “Programmed cell death protein 1 (PD-1) and programmed death-ligand 1 (PD-L1) inhibitors “are a promising class of drugs that have demonstrated effectiveness in the treatment of advanced NSCLC (1). These agents function by preventing PD-1 and PD-L1 from interacting., which are proteins expressed on immune cells and tumor cells, respectively. This interaction suppresses the immune system and allows cancer cells to evade immune surveillance. By blocking this interaction, PD-1 and PD-L1 inhibitors activate the immune system to target and destroy cancer cells.

PD-1 inhibitors, such as pembrolizumab and nivolumab, and PD-L1 inhibitors, such as durvalumab and atezolizumab, have been approved for the treatment of NSCLC based on the results of randomized clinical trials. These studies have shown that PD-1 and PD-L1 inhibitors can improve progression-free survival and overall survival in NSCLC patients compared to standard of care therapies (2–4). However, the effects of PD-1 and PD-L1 inhibitors on clinical imaging indices in lung cancer have not been well-established (5, 6).

To address this knowledge gap, we conducted a meta-analysis and systematic review of the literature to assess the effects of PD-1 and PD-L1 inhibitors on clinical imaging indices in lung cancer. Our primary research question was: What is the effect of PD-1 and PD-L1 inhibitors on clinical imaging indices in lung cancer? This review is important because understanding the effects of these treatments on clinical imaging indices can inform the use of these agents in clinical practice and guide future research.

There have been several meta-analyses and systematic reviews that have evaluated the effectiveness of PD-1 and PD-L1 inhibitors in the treatment of lung cancer. However, to our knowledge, this is the first meta-analysis and systematic review to specifically assess the effects of these treatments on clinical imaging indices in LC. Previous reviews have focused on outcomes such as overall survival and progression-free survival (2–5), while our review expands on this literature by examining the impact of PD-1 and PD-L1 inhibitors on clinical imaging indices such as tumor response and progression. Our review also includes a broader range of studies, including both randomized controlled trials and observational studies, whereas some previous reviews have only included randomized controlled trials. Overall, our review adds to the existing evidence on the use of PD-1 and PD-L1 inhibitors in LC by providing a comprehensive assessment of their effects on clinical imaging indices.

## 2 Methods

### 2.1 Literature search

The present study conducted a systematic search and meta-analysis of several databases including PubMed, Web of Science, Science Direct, and Scopus to identify relevant studies on the changes in clinical imaging-related indexes after PD-1/PD-L1 treatment of lung cancer. The search was conducted between 2011 and 2021 and included a variety of search terms, including “PD1-PD-L1 treatment of lung cancer, immune checkpoint inhibitors, programmed cell death 1, non-small cell lung cancer, programmed cell death ligand 1, Imaging and treatment-related adverse effects”. The research method used included the following search terms: “Imaging OR Radiology OR CT” and “adverse events OR irAEs” and “PD-1 inhibitors OR PD-1 treatment” and “Lung cancer”. The search was last conducted on October 15, 2021.

### 2.2 Inclusion and exclusion criteria

In this study, we conducted a systematic review to examine the effectiveness of PD-1 and PD-L1 inhibitors in the treatment of non-small cell lung cancer (NSCLC). We included single-arm or randomized trials evaluating these inhibitors as a single agent, as well as studies that used them in combination with other therapies. The inclusion criteria for the studies included in the review were: patients with advanced or recurrent NSCLC who had previously been treated with PD-1/PD-L1 monotherapy, patients who had successive imaging information for the evaluation of immune checkpoint inhibitors pneumonitis and objective responses, and patients treated with immune checkpoint inhibitors as monotherapy. We excluded studies that did not meet these criteria, such as those that included patients who received immunotherapy in combination with chemotherapy or had previous tuberculosis or fungi infectious diseases in the lung before immunotherapy.

### 2.3 Data extraction

The researchers conducted a thorough review of titles, abstracts, and full texts to extract data from each study for the purpose of analyzing imaging findings. The extracted data included the number of enrolled patients, mean or median age and sex of participants, distribution of lesions, and typical and less common abnormalities such as ground-glass opacities, consolidation, linear opacities, patchy shadow, air bronchogram, bronchial wall thickening or bronchiectasis, vascular enhancement, and pleural effusion. They also analyzed the diagnostic value of CT or CXR in the diagnosis of lung cancer if data was available in the studies.

### 2.4 Risk of bias assessment

The risk of bias was assessed using the New Castle-Ottawa scale, and the results are summarized in **Table 1**. Each study was evaluated based on its methodology, and the number of stars indicates the level of detail provided for that specific item.

**Table 1:** Assessment of articles Quality, Newcastle Ottawa Scale.

Study Name	Selection	Comparability					Outcome
	Representativeness of the exposed cohort	Selection of the non exposed cohort	Ascertainment of exposure	Demonstration that outcome of interest was not present at start of study	Assessment of outcome	Was follow-up long enough for outcomes to occur	Adequacy of follow up of cohorts
Francesco Alessandrino et al, 2019 (7)	*		*	*	*	*	*
Hyesun Park et al, 2020 (8)	*		*	*	*	*	*
Karthik Suresh et al, 2018 (9)	*		*		*	*	*
KENJI Nakahama et al, 2018 (10)	*		*			*	
Zongqiong Sun et al, 2021 (11)	*					*	
Tomoko Yamamoto Funazo et al, 2019 (12)	*					*	*
Daniel A. Smith et al, 2021 (13)	*				*	*	
Biagio Ricciuti et al, 2018 (14)	*		*			*	*
Tomohiro Itonaga et al, 2021 (15)			*	*	*	*	
Federica Ciccarese et al, 2021 (16)	*		*		*		
Myriam Delaunay et al, 2017 (17)							
Keigo Kobayashi et al, 2018 (18)	*						
Yuwen Zhou et al, 2020 (19)	*		*	*		*	
Shao et al., 2020 (20)	*		*		*	*	
Jun Fukihara et al, 2019 (21)	*		*	*	*	*	*
Pierpaolo Correale et al, 2020 (22)	*		*	*	*	*	
Xiangling Chua et al., 2020 (23)	*		*	*		*	

"*", high-quality choices.

### 2.5 Statistical analysis

In this study, meta-analysis was conducted using the-R Statistical Software. The prevalence of adverse effects related to radiologic findings in patients treated with PD-1 and PD-L1 inhibitors was analyzed using dichotomous data (events, total). The incidence and severity of immune-related adverse events were ranked in the extracted literature. The prevalence and 95% confidence interval of the frontline and subsequent therapy groups were estimated by dividing the number of observed follow-ups by the person-time follow-up. Heterogeneity was evaluated using Chi^2^ testing and the I^2^ statistic. A P value less than 0.05 indicated significant heterogeneity, and an I^2^ value greater than 50% was considered considerable heterogeneity.

## 3 Results

The study analyzed the incidence and types of immune-related adverse events (irAEs) in patients treated with immune checkpoint inhibitors (ICIs) for non-small cell lung cancer (NSCLC). A total of 2383 articles were identified through database searching, but only 17 of them were selected for analysis based on certain criteria (7–19, 21–24), such as the type of therapy used, the number of patients, and the duration of follow-up.

**Figure 1:**
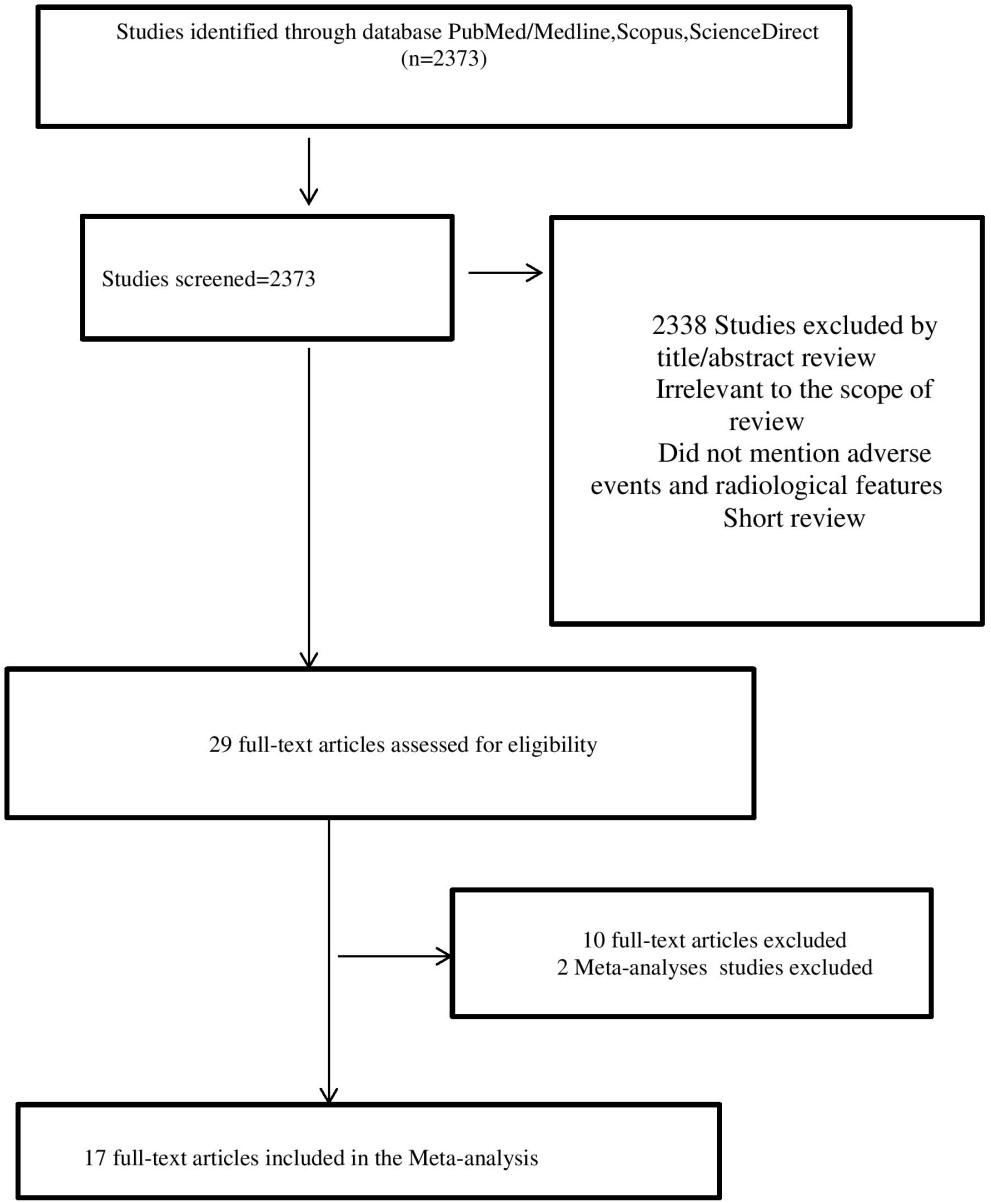
Flowchart of the Study Selection.

The baseline characteristics of the selected studies were grouped according to various factors, including the number of patients receiving first-line or subsequent ICI treatment, the type of therapy used, and the duration of follow-up (**Table 2**; **Figure 1**).

**Table 2:** Baseline of the Selected Studies.

Author- Year	First Time Treated with PD-1/PD-L1	Subsequent Therapy	Therapy	Age mean / median		Duration Of FollowUp(weeks) First time	Duration of Follow Up Subsequent Line
**1-Francesco Alessandrino-2019**	137	–	PD-1 inhibitor nivolumab monotherapy	65		5	–
**2-Hyesun Park-2020**	53	-	PD-1/PD-L1 monotherapy	65		12.1	–
**3-Karthik Suresh-2018**	-	199	All patients, including those in whom ICI therapy was discontinued	68		34.2	34.2
**4-Kenji Nakahama-2018**	201		Nivolmab	64		–	13.91
**5- Zongqiong Sun-2021**	98	-	PD-1/PD-L1 inhibitor monotherapy	59		156	–
**6-Tomoko Yamamoto Funazo-2019**	111	-	Nivolumab	68		9.7	–
**7- Daniel A. Smith-2021**	136	-	ICI	65		–	–
**8-Biagio Ricciuti-2018**	-	195	PD1 (nivolumab or pembrolizumab)	63		–	–
**9-Tomohiro Itonaga-2020**	29		PD-1 inhibitors	71		29	–
**10- Federica Ciccarese-2021**	82	–	ICI	68.5		–	–
**11-Myriam Delaunay-2017**	15	64	PD-L1/PD1	63		1	7
**12-Keigo Kobayashi-2018**	–	142	Nivolumab	67		–	–
**13- Yuwen Zhou-2020**	191	–	PD-1 inhibitors	58		9	–
**14-Satoshi Watanabe2019**	231		Nivolumab Pembrolizumab	66		34	–
**13-Keigo Kobayashi-2018**	–	142	Nivolumab	67		–	–
**14- Yuwen Zhou-2020**	191	–	PD-1 inhibitors	58		9	–
**15-Jun Fukihara2019**	170		Nivolumab or pembrolizumab monotherapy	67		–	
**16-Pierpaolo Correale-2020**	189	-	Received therapy with anti-PD-1 (nivolumab or pembrolizumab)	64		–	
**17-Xiangling Chua-2020**	102	189	ICIs	62		–	

"-", Not Applicable.

The incidence of irAEs was found to be more prevalent in patients receiving first-line treatment for NSCLC compared to those receiving subsequent treatments. This difference was statistically significant (p<0.001) for both high and low-grade irAEs. This means that the likelihood of experiencing an irAE was significantly higher for patients receiving first-line treatment compared to those receiving subsequent treatment.

The most common irAEs in the clinical studies were pneumonitis, thyroiditis, and colitis. Less common irAEs were hepatitis, pancreatitis and pancolitis.

The outcome data for the 17 included articles is presented in **Table 3**. This data provides further information about the characteristics of the studies and the patients included in the analysis.

**Table 3:** Outcoms data of the 17 included articles.

study ID	sample size	weight	first time treated wit PD-1/PD L1 (n)	Previously treated (n)	Male %	Age	GGO	Consolidation	Interlobular septal thickening	Pneumonitis	Colitis	Hepatitis	Pancreatitis	Pancolitis	SCAD	Thyroiditis
Francesco Alessandrino et al, 2019 (7)	137	5%	137	-	45	65	-	-	-	-	18	2	-	14	2	-
Hyesun Park et al, 2020 (8)	53	2%	53	-	51	65	4	4	-	10	9	1	1	7	2	3
Karthik Suresh et al, 2018 (9)	205	8%	6	199	56	68	31	33	8	39	-	-	-	-	-	-
KENJI naKAHAMA et al, 2018 (10)	201	8%	201	-	68	68	-	-	-	-	-	-	-	-	-	-
Zongqiong Sun et al, 2021 (11)	98	4%	98	-	58	59	5	-	-	18	-	-	-	-	-	-
Tomoko Yamamoto (12) Funazo et al, 2019 (11)	111	4%	111	-	66	68	-	-	-	-	1	21	-	-	-	-
Daniel A. Smith et al, 2021 (12)	136	5%	136	-	45	65	-	49	-	14	-	-	-	-	-	-
Biagio Ricciuti et al, 2018 (13)	195	8%	na	195	66	63	-	-	-	-	21	-	-	-	-	-
Tomohiro Itonaga et al, 2021 (15)	29	1%	29	-	80	71	-	-	-	-	-	-	-	-	-	20
Federica Ciccarese et al, 2021 (16)	82	3%	82	-	66	68.5	3	-	-	5	3	-	1	-	-	-
Myriam Delaunay et al, 2017 (17)	79	3%	15	64	64	63	52	34	10	30	-	-	-	-	-	-
Keigo Kobayashi et al, 2018 (18)	142	6%	na	142	79	67	-	-	-	-	4	-	-	-	-	15
Yuwen Zhou et al, 2020 (19)	191	7%	191	-	72	58	-	-	-	-	-	-	-	-	-	24
Shao et al., 2020 (20)	231	9%	231	-	79	66	16	-	-	16	-	-	-	-	-	-
Jun Fukihara et al, 2019 (21)	170	7%	170	-	74	67	25	-	-	4	-	-	-	-	-	-
Pierpaolo Correale et al, 2020 (22)	189	7%	189	-	80	64	29	-	-	29	-	-	-	-	-	-
Xiangling Chua et al, 2020 (23)	300	12%	102	198	80	62	2	-	-	45	-	-	-	-	-	-

"-", Not Applicable.

**Figure 2:**
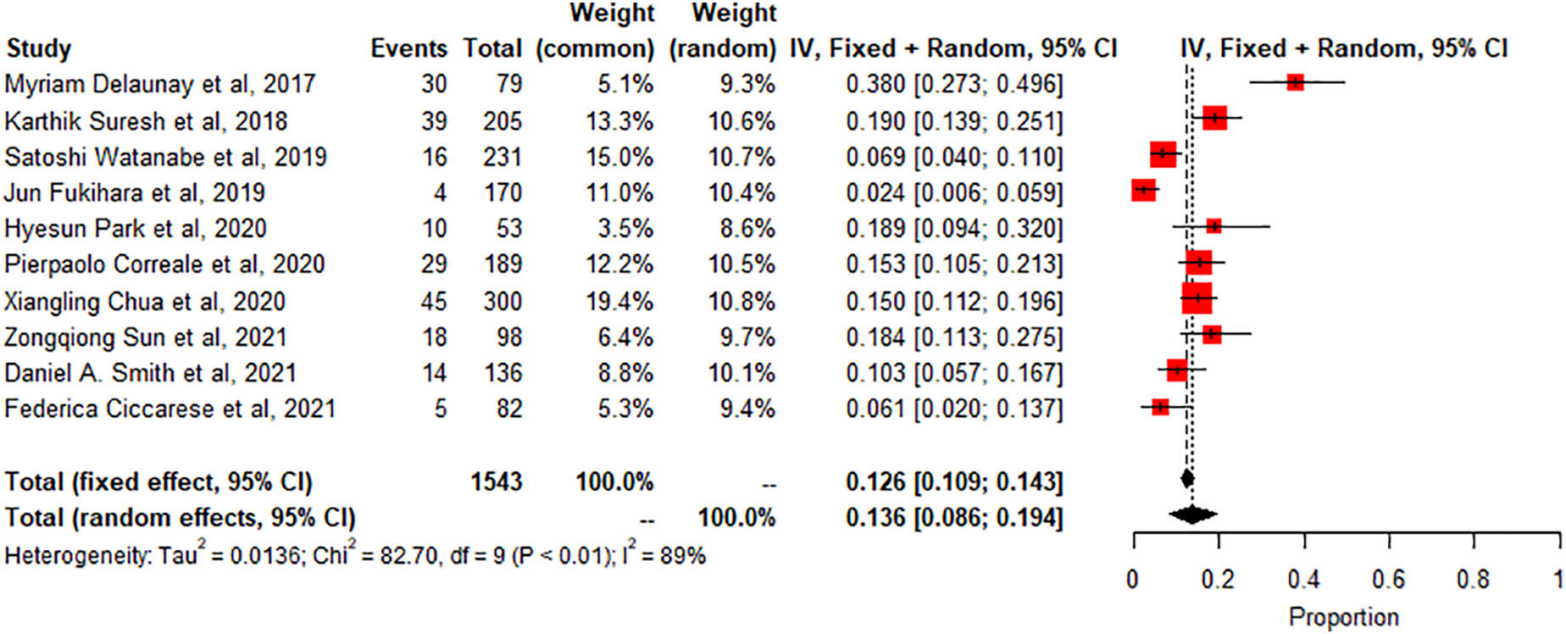
Shows a meta -Analysis of 10 studies of pneumonitis related radiologic findings.

**Figure 3:**
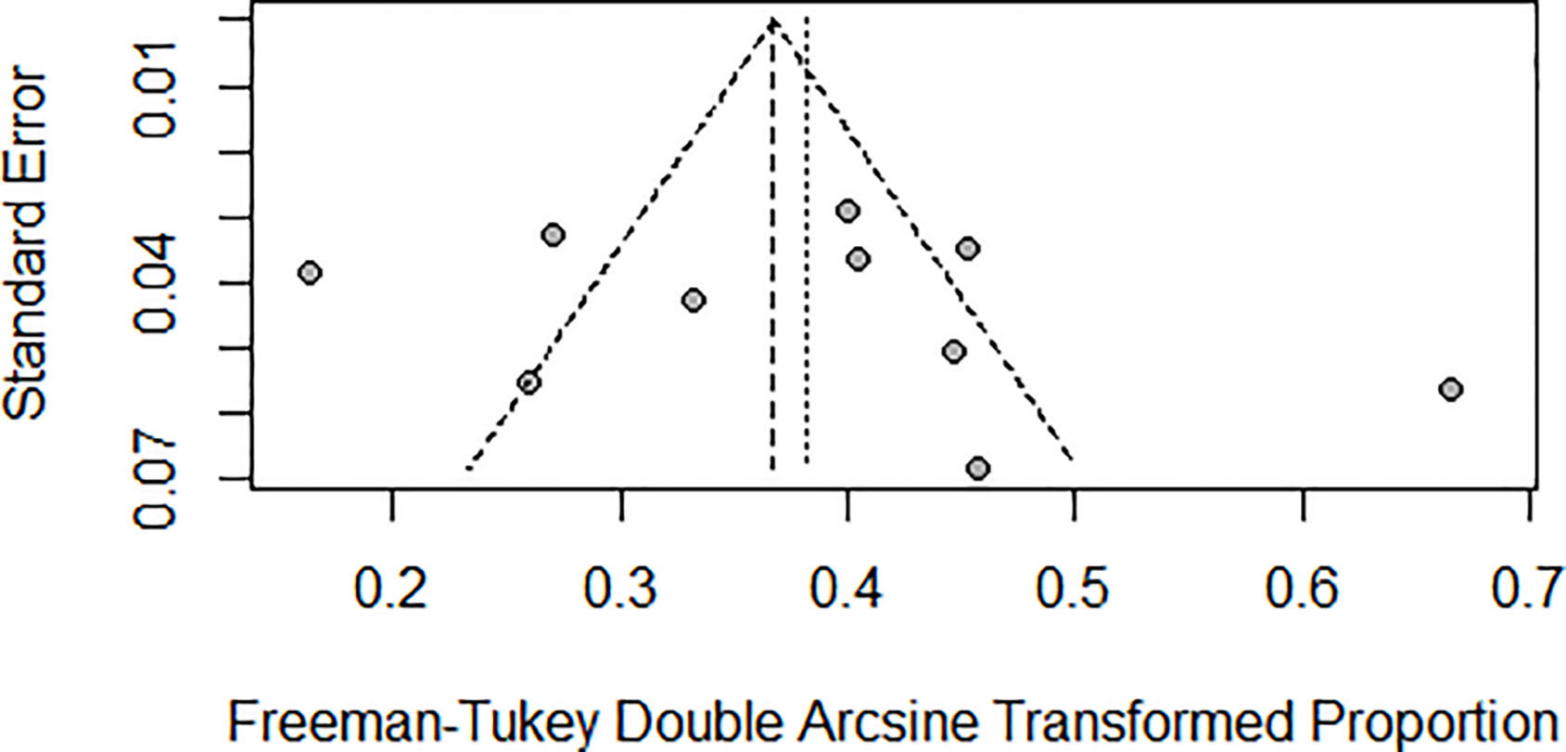
Shows the Pneumonitis funnel plot.

The study also looked at the prevalence of specific irAEs, such as pneumonitis, colitis, and SCAD, in different subgroups of patients. The results were as follows:

Pneumonitis: A meta-analysis of 10 studies (n=1543) shows prevalence of pneumonitis-related adverse effects, The degree of heterogeneity in the interpretation of the forest plot is high, as indicated by the I2 value of 89.1% and the significant p-value in the test of heterogeneity (p < 0.0001) **Figure 2**. Possible publication bias was determined by visual inspection of a funnel plot **Figure 3** and the egger test conducted **Table 4**.

**Table 4:** Linear regression test of funnel plot asymmetry.

	Z	p
**sei**	**0.74**	**0.47**

The table shows Regression test for Funnel plot asymmetry (“Egger’s test”).

**Figure 4:**
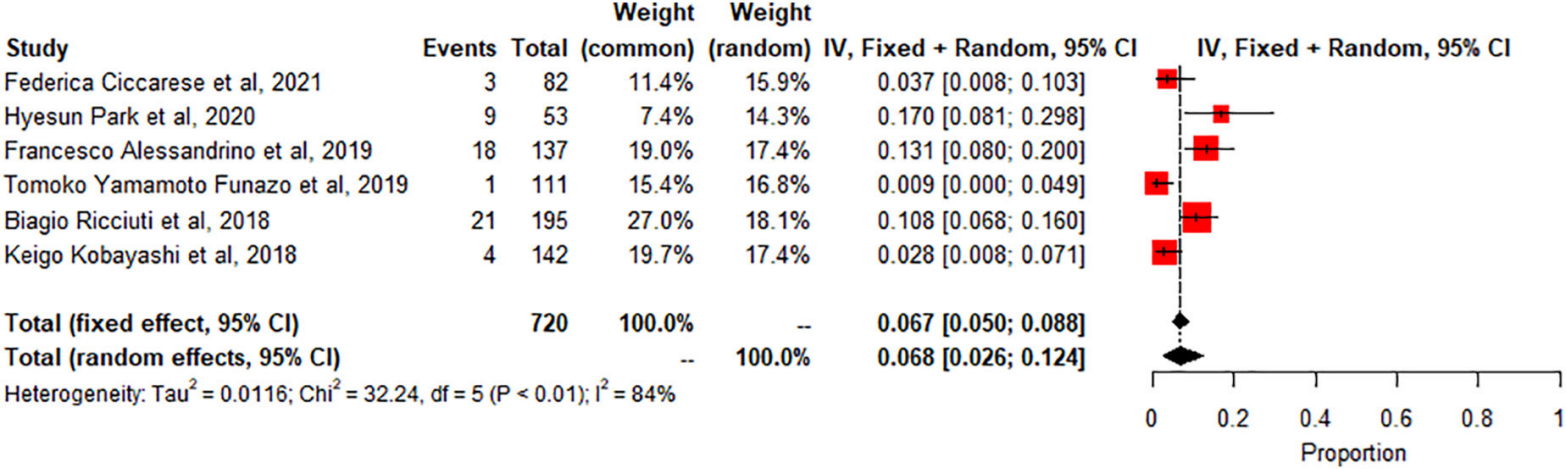
A meta -analysis of colitis-related radiologic findings.

Colitis: A meta-analysis of 6 studies (n=720) showed the pooled prevalence of colitis-related adverse effects The degree of heterogeneity in the interpretation of the forest plot for colitis is substantial, as indicated by the I2 value of 84.5% and the significant p-value in the test of heterogeneity (p < 0.0001). **Figure 4**.

**Figure 5:**
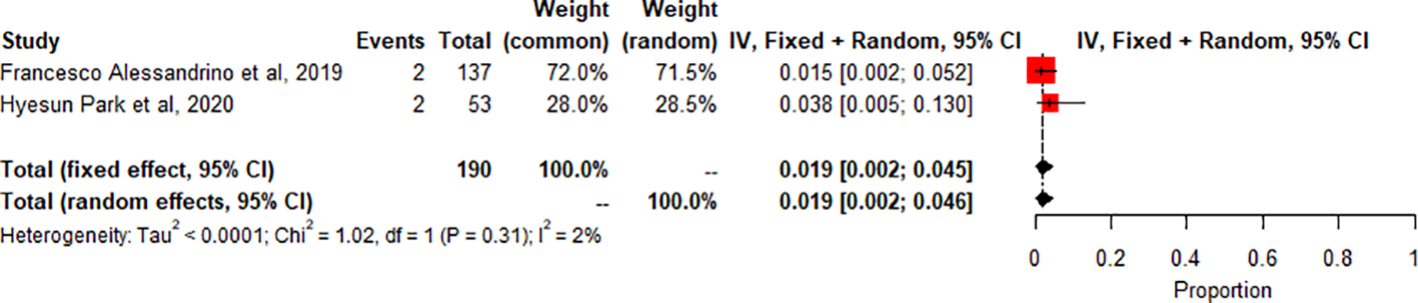
A meta -Analysis Of SCAD related radiologic findings.

Segmental Colitis associated with diverticulosis (SCAD): A meta-analysis of 2 studies (n=190) showed the pooled prevalence of SCAD-related adverse effects compared to controls or other subgroups. SCAD forest plot shows low heterogeneity (I2 2.2%, p=0.3119). Small, significant effect size seen (0.0187 common effect, 0.0188 random effects, 95% CI: 0.0024 to 0.0461). **Figure 5**.

**Figure 6:**
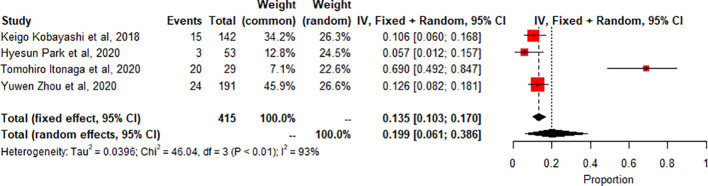
A meta -Analysis Of thyroiditis-related radiologic findings.

Meta-analysis of thyroiditis prevalence shows high heterogeneity (I2 93.5%, p<0.0001). 4 studies (n=415, 62 events) combined, proportion 0.1989 (95% CI [0.0611, 0.3864]) using random effects model **Figure 6**.

Other irAEs (e.g., hepatitis, pancreatitis): No statistical analysis was presented for these irAEs, so it is not clear how the prevalence of these events compared to controls or other subgroups.

Age and prior treatment with radiotherapy were found to be determinant factors in the overall association with irAEs. This means that these factors may have influenced the likelihood of experiencing an irAE.

One study indicated a high incidence of checkpoint inhibitor pneumonitis, although it is not clear how this incidence compares to other studies or subgroups.

## 4 Discussion

The main findings of the study were that the incidence of irAEs was more prevalent in patients receiving first-line treatment for NSCLC compared to those receiving subsequent treatment and that the most common irAEs were pneumonitis, hypothyroidism, hyperthyroidism, and skin reactions. This suggests that the likelihood of experiencing an irAE may be higher in patients receiving their first course of ICI treatment for NSCLC. This finding is consistent with previous research on the incidence of irAEs in patients treated with ICIs, which has also found a higher incidence in patients receiving first-line treatment compared to those receiving subsequent treatment (8, 20).

The study also found that the prevalence of specific irAEs, such as pneumonitis, colitis, and SCAD, varied across different subgroups of patients. For example, the meta-analysis of 10 studies (n=1543) found a significantly higher prevalence of pneumonitis-related adverse effects compared to controls or other subgroups (p<0.01, statistically significant heterogeneity with I2 testing at 89%). This means that the likelihood of experiencing pneumonitis as an irAE was significantly higher in the subgroup of patients included in these ten studies compared to controls or other subgroups. On the other hand, the meta-analysis of 2 studies (n=190) found a low prevalence of SCAD-related adverse effects compared to controls or other subgroups (p>0.01, statistically non-significant heterogeneity). This indicates that the likelihood of experiencing SCAD as an irAE was not significantly different in the subgroup of patients included in these two studies compared to controls or other subgroups. The incidence of thyroiditis was also high in many trials, as reported by Park et al. (2020), with many patients presenting with thyrotoxicosis and subsequently developing hypothyroidism (8). Thyroiditis is hard to diagnose because sometimes it presents as a normal progression of cancer, and in some cases, it is entirely asymptomatic. If the patient is not closely monitored or thyroid-stimulating hormone (TSH) is not checked regularly, the symptoms may go unnoticed, leading to severe, sometimes irreversible consequences due to under diagnosis. Therefore, clinicians must stay vigilant and make sure patients continue with clinical imaging to detect the slightest change in thyroid function to manage the side effects better. Immune-related thyroiditis is seen via Positron Emission Tomography, abbreviated as (PET) CT, and presents as new fluorodeoxyglucose (FDG) uptake in the thyroid gland. Park et al. (2020) noted that even though the routine CT scans are not very specific, there was a change in the heterogeneous enhancement of the gland when immune checkpoint therapy was started, which calls for further evaluation (8).

The finding that the prevalence of specific irAEs varies across different subgroups of patients highlights the importance of considering patient-specific factors in the management of irAEs. For example, clinicians may need to be more vigilant in monitoring and managing irAEs in patients at higher risk of experiencing these events, such as those with a higher prevalence of pneumonitis.

In addition to the type of ICI treatment received and the specific type of irAE, the study found that age and prior treatment with radiotherapy were determinant factors in the overall association with irAEs. This suggests that these factors may influence the likelihood of experiencing an irAE and should be considered in the management of these events.

One study included in the analysis indicated a high incidence of checkpoint inhibitor pneumonitis (23), although it is not clear how this incidence compares to other studies or subgroups. Pneumonitis is a potentially serious irAE that can have significant impacts on treatment outcomes and patient quality of life. Therefore, it is important for clinicians to be aware of the potential for pneumonitis in patients treated with ICIs and to implement appropriate monitoring and management strategies.

Overall, the results of this study highlight the importance of considering the potential for irAEs in patients treated with ICIs for NSCLC and the need for careful monitoring and management of these events. The finding that the incidence of irAEs is higher in patients receiving first-line treatment and that the prevalence of specific irAEs varies across different subgroups of patients underscores the need for personalized approaches to the management of irAEs, taking into account the unique characteristics and needs of each patient. Future research should focus on identifying strategies to mitigate the impact of irAEs on treatment outcomes and improve the quality of life of patients experiencing these events.

The role of imaging in the management of irAEs was also discussed in the article. All imaging studies included in the analysis were performed after nivolumab discontinuation and before starting subsequent therapy and were included as irAEs can occur months or even years after treatment discontinuation. One study example had patients developing irAEs that were radiologically notable, with an average onset time of about seven weeks. The study found that imaging can be useful in the diagnosis and management of irAEs, as it can help to identify specific patterns and changes associated with these events.

## 5 Limitations

In the examination of the results, it is essential to consider several limitations inherent in the present study. Primarily, the majority of the studies incorporated in this review were retrospective cross-sectional investigations, which hold the potential to introduce biases into the outcomes. This potential bias was evidenced by the funnel plot and Egger’s test results, suggesting the presence of publication biases. Moreover, the studies demonstrated considerable heterogeneity, which may impact the generalizability of the findings. Thus, when interpreting the results, it is crucial to account for the possibility of heterogeneity, given the nature of the studies included in this review. Despite these limitations, the analysis sheds light on various aspects of the research question and provides valuable insights for future investigations.

## 6 Conclusion

The present study provides important insights into the incidence and types of irAEs in patients treated with ICIs for NSCLC and the role of imaging in the management of these events. These results highlight the need for careful monitoring and management of irAEs to ensure the best possible outcomes for patients treated with ICIs for NSCLC.

## Data availability statement

The original contributions presented in the study are included in the article/supplementary material. Further inquiries can be directed to the corresponding authors.

## Author contributions

NM and ZX conceived the presented idea, while RZ and E-HX developed the theoretical framework. NM and SM performed the analytical methods. All authors contributed to the discussion of the results and the final manuscript, and provided their approval of the submitted version.
